# The Use of Computational Geometry Techniques to Resolve the Issues of Coverage and Connectivity in Wireless Sensor Networks

**DOI:** 10.3390/s22187009

**Published:** 2022-09-16

**Authors:** Sharmila Devi, Anju Sangwan, Anupma Sangwan, Mazin Abed Mohammed, Krishna Kumar, Jan Nedoma, Radek Martinek, Petr Zmij

**Affiliations:** 1 Department of Computer Science & Engineering, Guru Jambheshwar University of Science & Technology, Hisar 125001, India; 2 College of Computer Science and Information Technology, University of Anbar, Anbar 31001, Iraq; 3 Department of Hydro and Renewable Energy, Indian Institute of Technology, Roorkee 247667, India; 4 Department of Telecommunications, Faculty of Electrical Engineering and Computer Science, VSB-Technical University of Ostrava, 70800 Ostrava, Czech Republic; 5 Department of Cybernetics and Biomedical Engineering, Faculty of Electrical Engineering and Computer Science, VSB-Technical University of Ostrava, 70800 Ostrava, Czech Republic; 6 Industrial Engineering—Brose Group, Prumyslovy Park 302, 74221 Koprivnice, Czech Republic

**Keywords:** computational geometry, convex hull, Delaunay Triangulation, Voronoi Diagram, Voronoi Tessellation, wireless sensor networks

## Abstract

Wireless Sensor Networks (WSNs) enhance the ability to sense and control the physical environment in various applications. The functionality of WSNs depends on various aspects like the localization of nodes, the strategies of node deployment, and a lifetime of nodes and routing techniques, etc. Coverage is an essential part of WSNs wherein the targeted area is covered by at least one node. Computational Geometry (CG) -based techniques significantly improve the coverage and connectivity of WSNs. This paper is a step towards employing some of the popular techniques in WSNs in a productive manner. Furthermore, this paper attempts to survey the existing research conducted using Computational Geometry-based methods in WSNs. In order to address coverage and connectivity issues in WSNs, the use of the Voronoi Diagram, Delaunay Triangulation, Voronoi Tessellation, and the Convex Hull have played a prominent role. Finally, the paper concludes by discussing various research challenges and proposed solutions using Computational Geometry-based techniques.

## 1. Introduction

Wireless Sensor Networks (WSNs) have attracted considerable global attention recently. These networks enwrap tiny devices, commonly known as sensor nodes. Although these devices have very limited energy and a slow processing speed, they can sense and transmit the collected data from the required area of interest to the sink node for processing. The sensor network life span can be described as the time span for which the sensing task is performed by the network nodes and the time span for which the data is transmitted towards the base station. Due to the hardware disruption of power exhaustion, certain transmitting nodes become unreachable during this phase. The applicability of WSNs has been seen in various fields depending on the working arena; these include: military, security, healthcare, environment, target detection, and data collection. It is pertinent to mention that certain unique qualities are linked to various applications, each requiring specific features from a WSN solution [1,2]. WSNs can be categorized as Terrestrial WSNs, Underwater WSNs, Mobile WSNs, Underground WSNs, and Multimedia WSNs [3,4]. Various redundant issues commonly seen in WSNs are related to coverage and connectivity. Organizing a limited number of sensors optimally in a complex networking environment is also a challenging task. In order to solve these issues in existing literature, many approaches have been proposed to date. The ongoing research on coverage and connectivity improvement has overwhelmingly generated a lot of interest in new researchers [3,5,6,7,8,9,10]. The key focus is on designing and developing energy-efficient networks with a minimum cost.

For an efficient working model, the Prioritized Geometric Area Coverage (PGAC) approach to improve the coverage area based on the Voronoi Diagram is discussed in [11]. Voronoi cells are classified as smaller, medium, and larger cells based on the working direction of the sensor nodes. The working direction of the sensor is selected on the basis of the relationship between the sensor’s covered area and the cell area. This approach benefits from the reduced overlapped area and the increased network lifetime. For better scalability, an energy-efficient hole detection algorithm based on Delaunay Triangulation is given in [12]. In this approach, triangles are categorized as acute, obtuse, and right-angled triangles based on the angles of the Delaunay Triangulations. Efficient deployment of nodes leads to network optimization and directly impacts the overall performance of WSNs. To enhance the target localization accuracy, a Delaunay Triangulation-based method is discussed in [13]. This localization algorithm can also be upgraded from a two-dimensional to a three-dimensional co-ordinates system. For the accurate estimation of the target’s location, the geometric structures such as the triangle or the tetrahedron are identified firstly in the neighborhood of the target. Then, a localization algorithm is used to find the co-ordinates of the target’s location. The dimensions upgrading from two-dimensional to three-dimensional are very useful for practical applications. As a limitation of the proposed work, authors have also suggested verifying the network lifetime and the delay of the proposed algorithm in real-world scenarios for both the two-dimensional and three-dimensional cases. Two algorithms, namely Delaunay Triangulation with moving sensors and the Convex Hull with moving sensors, for the detection and boundary refinement of Intangible Continuous Objects are described in [14]. Firstly, sensors are grouped into covered and uncovered sensor sets. Then, the concepts of Delaunay Triangulation and the Convex Hull are applied to static sensors to determine the rough boundary of the Intangible Continuous Objects. For refinement of the enclosed area of the Intangible Continuous Objects, a moving mechanism is introduced to the sensors in order to adjust their positions. The results show that 135–157% and 102–145% of the actual area of the Intangible Continuous Objects is covered by Delaunay Triangulation with moving sensors and the Convex Hull with moving sensors, respectively. In order to resolve the fundamental issue of node’s positions a Voronoi diagram-based scheme is discussed in [15]. First of all, the whole area is partitioned into several small regions using a Voronoi diagram. Then, to optimize and accurately estimate the position of the target node, a support vector machine is used. The proposed scheme is compared with the Optimal Region Selection Strategy based on the Voronoi and the Weighted Voronoi diagrams. The results show that the proposed localization scheme is better than both of the above-mentioned strategies.

This paper contributes through the following aspects:

- The present study introduces effective methods and ideas for accomplishing a constructive system for modeling WSNs;
- With the help of scientometric analysis, this paper throws light upon the significance of Computational Geometry (CG)-based techniques and their utilization in WSNs;
- It sheds light on the critical role of Computational Geometry-based techniques in addressing issues related to coverage and connectivity, which are considered to be inherent problems of WSNs;
- This opens a new frontier for future research scholars to address the problems of coverage and connectivity holes efficiently and strategically, based on the Computational Geometry techniques in WSNs.

The primary goals of this study are to answer some relevant questions given as follows:

− Q1. How is the issue of coverage and connectivity in WSNs for two-dimensional or three-dimensional networks addressed?
− Q2. How can coverage and connectivity in Directional Senor Networks be improved?
− Q3. How can the issue of coverage and connectivity within the mobile environment (where sensor nodes, target points or both can be in motion) and the heterogeneous environment be addressed?
− Q4. How can the issue of coverage and connectivity when obstacles are present in the environment be addressed?
− Q5. How efficiently can the discovery and healing of coverage and communication holes can be achieved?
− Q6. How perfectly can energy resources be managed?
− Q7. How can the issue of coverage and connectivity on the boundaries of the area of interest be resolved?

This paper briefly recapitulates the importance of CG-based Techniques in WSNs. Section 2 defines the connection between Computational Geometry and WSNs and Section 3 illustrates the role of CG Techniques in improving Coverage and Connectivity in WSNs. Section 4 describes the present research challenges and their solution with the help of CG Techniques. Lastly, Section 5 concludes the paper and provides the scope for future studies.

## 2. Computational Geometry and WSNs

### 2.1. Computational Geometry (CG)

Computational Geometry arose in the late 1970s from the field of algorithms design and analysis. It focused on various recurring problems arising from scientific computing, geographic information systems, robotics, computer graphics, and recent scenarios that have gained interest in wireless networks during the nascent stages, wherein geometric algorithms might play a crucial role. Geometric algorithms are developed explicitly to analyze the structural properties and to explore the inclusion and exclusion relationships between two points or hyperplanes (or both). The Convex Hull, the Voronoi Diagram, Triangulation (Regular, Delaunay, and so on), the Hyperplane arrangement, and the Intersections are just a few structural properties [16]. Computational Geometry describes various geometrical objects such as points, line segments, and polygons. The same can be applied to overcome the famous problem in the art gallery, where at least one of the security guards observes the boundary of the gallery [17]. There are a variety of data structures related to Computational Geometry. Some essential data structures are the Voronoi Diagram, Delaunay Triangulation, the Convex Hull, the Largest Empty Circle, the Euclidean Shortest Path, Polygon Triangulation, and the Closest Pair of Points, etc. However, in WSNs, data structures like the Voronoi Diagram, Delaunay Triangulation, Voronoi Tessellation, and the Convex Hull have been used infrequently in recent years. These promising Computational Geometric data structures play a crucial role in node deployment, coverage hole detection, and the healing process.

### 2.2. The Significance of CG Techniques in WSNs

**Figure 1:**
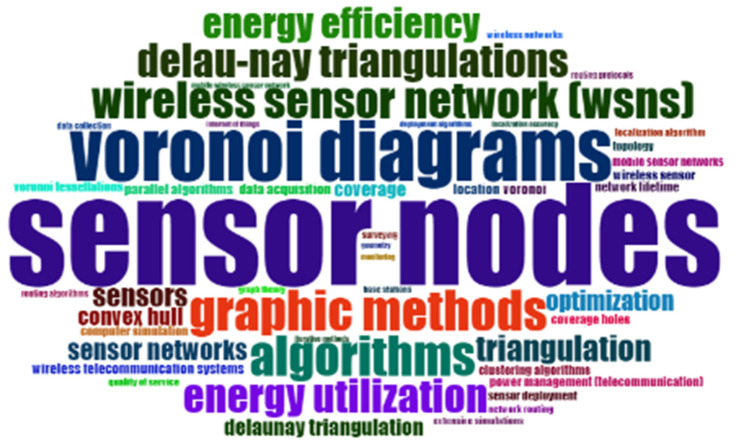
A word cloud of keywords.

It is pertinent to mention that network design for a wireless sensor is challenging. An efficient network needs to address a few important issues like limited batteries and memories, fault tolerance, dimensionality, and node location. Figure 1 shows the word cloud of keywords and the size of each keyword determines its importance in the given field.

The authors in [16] have described the use of a network topology control that can be constructed locally with a subgraph of the unit disk graph created with the underlying sparse features. The topology control in WSN aims at three things: i.e., maintenance of network connectivity, optimizing network lifetime, and designing power-efficient routing. The authors have also focused on the power expansion factor of numerous sparse geometric structures to be used as the network topology of WSN. It includes geometric structures such as the Low-Weight Structures, the Planar Structures, the Bounded Degree Planar Structures, the Bounded Degree structures, and the Bounded Degree Planar, among others. In WSN, the most common failure observed is an individual node failure. Hence, a fault-free network topology should have multiple-path connectivity between two sensors, including k-connectivity (k ≥ 1).

The authors in [18] have elaborated 14-connectivity and 6-connectivity among nodes by implementing two Voronoi Polyhedrons to maintain a fault-free network: two Voronoi Polyhedron Truncated Octahedron and Hexagonal Prisms to maintain a fault-free network.

The Voronoi Diagram, Voronoi Tessellation, and Delaunay Triangulation are some of the most commonly used geometric structures. These geometric structures facilitate viable solutions for WSN problems such as Coverage assessment, reducing overall memory requirements, avoiding transmission of superfluous communication messages, and network optimization. Also, it is deliberately discussed in [5] that the Voronoi Diagram and the power diagram are also used as solutions for coverage problems in d-dimension (d ≥ 1) WSNs. The authors in [19] have proposed 3D sensors deployment based on Voronoi Tessellation to solve the coverage problem. The authors in [20] have presented a method based on a localized Voronoi Diagram to solve the localization problem in sensor nodes. Directional antennas can provide the necessary location information about neighboring nodes. This algorithm reduces the cost of location-finding devices and power consumption because it does not require a global positioning system.

### 2.3. Scientometric Analysis of CG and WSN

The scientometric analysis mainly focuses on the scientific study of available literature. This scientometric analysis aims to visualize the impact of the published literature time. This can be done using the information from the literature records such as the keywords, the authors, the countries, and the references. In the present study, the scientometric analysis assesses terms like computational geometry, sensor network, wireless sensor network, coverage, connectivity, and many more related words in the same domain. This scientometric analysis aims to visualize the impact of published literature over time. With this analysis, researchers can trust how influential the core area of their associated research is. Table 1 gives the preliminary information on which this analysis is performed.

**Figure 2:**
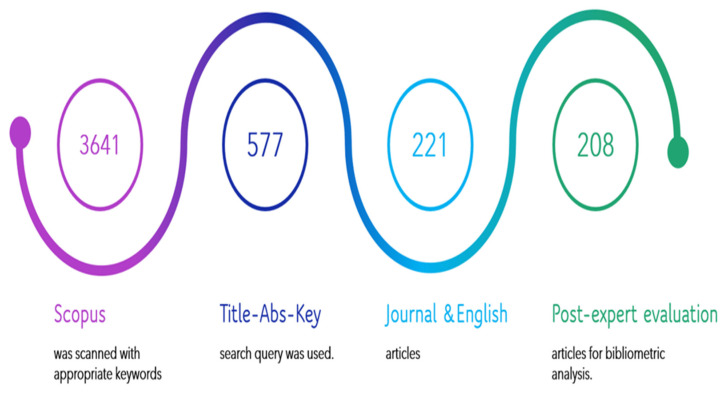
The Article Selection Process.

The process of article selection needed for the present study to perform scientometric analysis is represented by Figure 2.

This analysis covers (i) how the development of related terms, methods, and techniques cover different countries, (ii) how these are growing with time, and (iii) which authors are associated with, or working with, these terms, methods, and techniques, etc. The information presented in Table 1 has been extracted from the Scopus database and processed using the VOSviewer-Visualizing scientific landscapes, which is basically an online tool to construct and visualize bibliometric networks.

**Figure 3:**
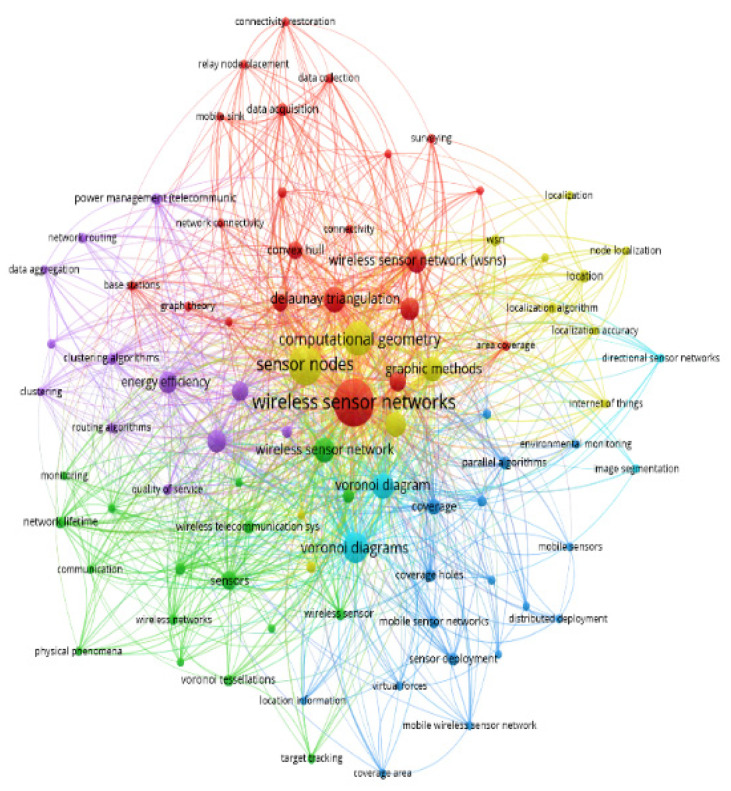
The network visualization of co-occurrence of all keywords using the VOSviewer.

Figure 3 presents the complex web of related keywords based on the co-occurrence of all keywords. Each sphere defines a keyword and its size is proportional to its occurrence. Therefore, the bigger sphere size represents a higher occurrence of the keyword. The different colors in the given figure divide all keywords into their sub-domains. Also, these sub-domains are interconnected with each other either directly or indirectly. The given figure shows how all keywords are bound to each other. This inter and intra-boundedness of keywords leads work with new dimensions, in the same and in interdisciplinary domains, to proceed further.

**Figure 4:**
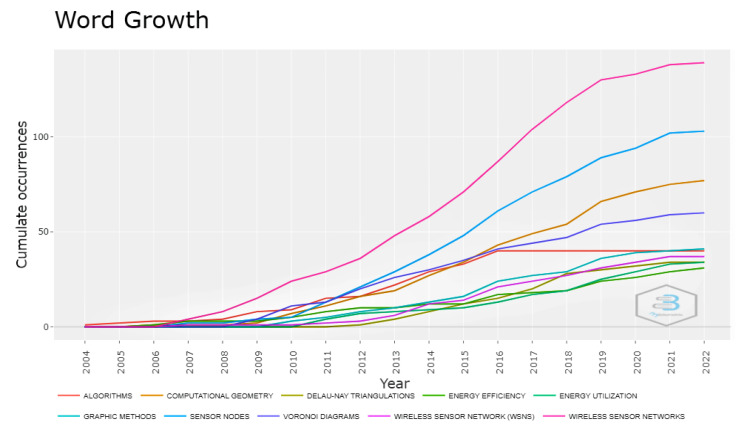
Word growth over time during the period 2004–2022.

Figure 4 shows how related terms such as Wireless Sensor Networks, Sensor Nodes, Computational Geometry, and Delaunay Triangulations, etc., are growing over time. The term Wireless Sensor Networks shows the highest growth in the given period. Other keywords such as Computational Geometry and Delaunay Triangulations also show a rapid growth in the defined period. This rapid growth shows the potential significance of Computational Geometry, other related terms, and WSNs to take the research to new heights in the given area.

**Figure 5:**
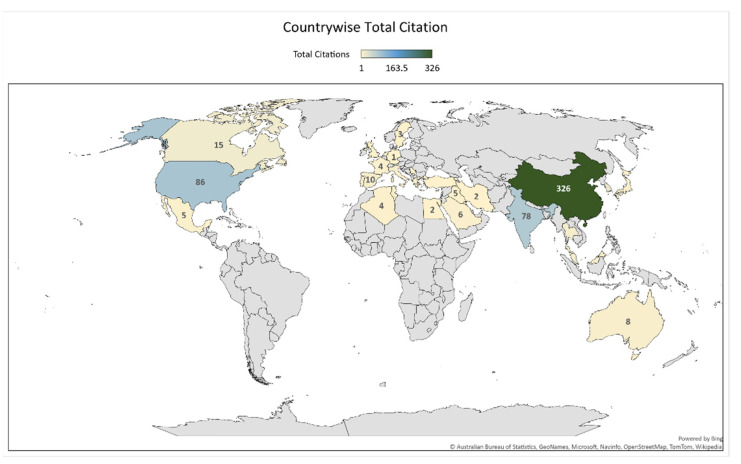
The total citations per country.

Figure 5 gives the total citations of given terms as contributed by each country. China is in the first rank as shown by the dark green color with a citation score of 326; followed by the USA and India (with citation scores of 86 and 78, respectively) as shown by a different shade of light blue. The other countries with a lower citation score are shown by a light dust color. The citation outcome reflected by China shows how extensively researchers in the country are working in this domain. China’s continued advancement in research is aided by four factors: a high population density and human capital base; a labor market that rewards academic meritocracy; a significant diaspora of scientists of Chinese origin; and a centralized government that is eager to fund science [21]. Also, this is a valuable indication for researchers worldwide that there is much scope for work associated with this domain.

**Figure 6:**
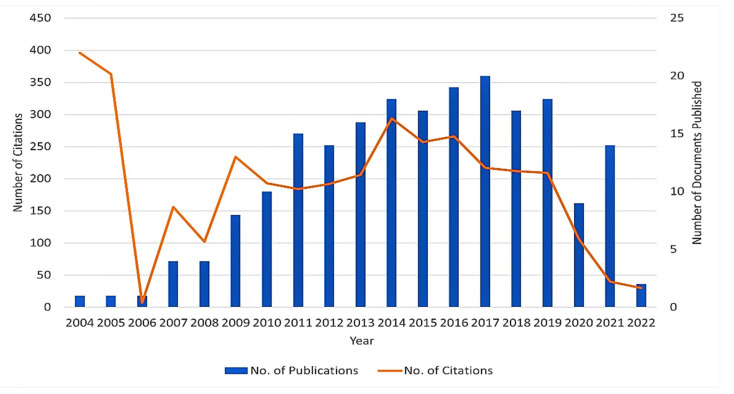
Documents and citations published yearly.

Figure 6 provides a two-way graph showing the yearly publication of the documents and respective citation counts relating to keywords chosen for this study. From 2004 to 2005, articles in this domain were frequently cited and 2017 is considered the year of the highest number of published documents based on the information obtained from the Scopus database in a specified time zone.

**Figure 7:**
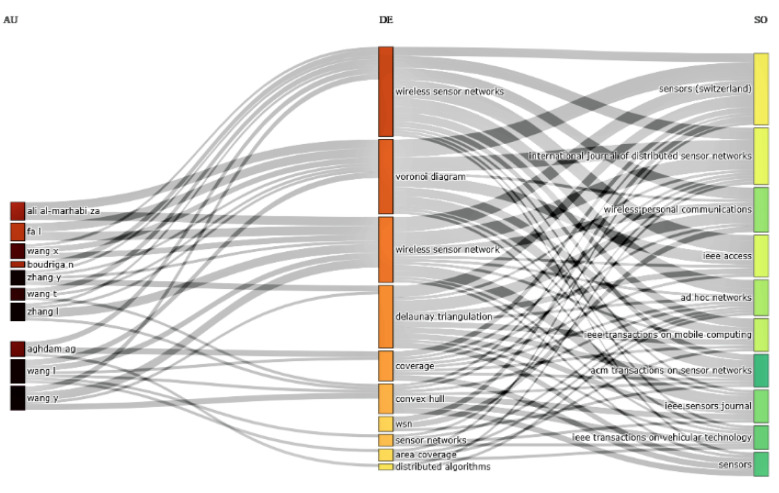
A three-field plot of authors, keywords, and publication sources.

Figure 7 shows a three-field plot of associated authors, keywords, and publication sources in this domain. The figure displays the ten most influential authors and their publication sources as reputable journals, along with the most frequently used keywords. This study shows that the keywords: Wireless Sensor Networks, Voronoi Diagram, Delaunay Triangulation, coverage, and Convex Hull are the significant keywords and that Wang I., Wany Y., Zhang I., and Ali Al-Marhabi Za are the authors who have mainly used these keywords. Moreover, *Sensors*, *International Journal of Distributed Sensor Networks*, *Wireless Personal Communication*, and *IEEE Access* are the major journals which are publishing the many articles in this domain.

### 2.4. Coverage and Connectivity in WSNs

The efficiency of WSNs largely depends on two main aspects, i.e., coverage and connectivity. The reliability factor of WSNs is guaranteed by these having full coverage in a given area of interest. Coverage falls into two broad categories: the area coverage and the target coverage. A particular region is covered by placing sensor nodes in the area coverage. On the other hand, a specific target region is covered by the pre-determined coordinates or positions in the target coverage. To enhance the coverage naturally, the phenomenon of m-coverage has been introduced wherein at least the ‘m’ sensors cover each target. It acts like a fail-proof system in which the target remains covered until the time the m-1 devices fail. A fully connected network has all the nodes communicating with each other either directly in a one-to-one fashion or via relay nodes. In WSNs, the connectivity is modeled using a graph. A network is k-connected if it contains at least k edge-disjoint paths connecting any pair of nodes at any point in time. Similarly, the network is connected until the time that the k-1 devices fail. The WSN coverage strategies are divided into three main categories: self-scheduling, meta-heuristic, and classical deployment. The classical deployment techniques are further categorized into Computational Geometry, Force-based, and Grid-based techniques. 

The authors in [5] have explained the construction of minimal-connected cover sets to solve coverage and connectivity problems and enhance QoS in WSNs. The fundamental concept is to compute and then connect a cover set. The authors also discussed an algorithm that identifies fully supported sensor nodes without error with the help of Voronoi Tessellation. Such supported sensor nodes can be recognized by estimating the coverage redundancy of the Voronoi cells. The Voronoi partition and the Clique partition are used to predict the mobility of sensor nodes to get the desired network connectivity and target coverage. One of the major goals of future research is to reduce the number of sensor nodes deployed in a particular area while maintaining the network connectivity and the coverage of the target area [17].

## 3. CG Techniques for Improving Coverage and Connectivity in WSNs

In WSNs, Computational Geometry plays an essential role in enhancing the Quality of Service (QoS) of several sensor nodes that can be optimized using new computational techniques; and the longevity of WSNs can be increased to some extent. Coverage holes can be formed in the target field due to the inherent nature and the random deployment of WSNs. Certain strategies must be used to guarantee that the area of interest is entirely free of any coverage flaws. Furthermore, Computational Geometry is an essential strategy for efficiently detecting and healing coverage holes, enhancing network quality, and extending the network’s life. The geometric distribution of sensors considerably affects the design of WSNs.

### 3.1. The Voronoi Diagram-Based Technique

**Figure 8:**
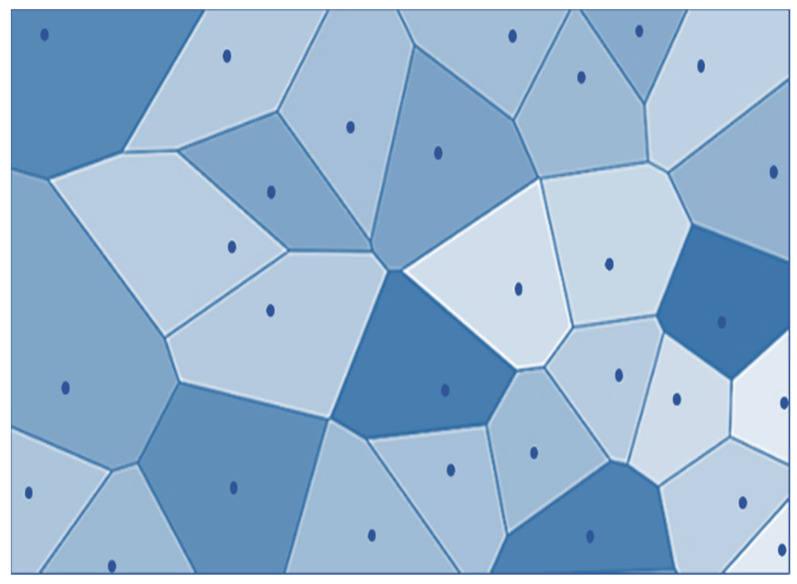
A Voronoi Diagram.

In Computational Geometry, Voronoi Diagrams are integral data structures. They regenerate a specific region into many regions with the help of generators (points). A dominant part represents each point, which is termed a Voronoi cell, consisting of all topics closer to it than any other issue, as shown in Figure 8.

**Figure 9:**
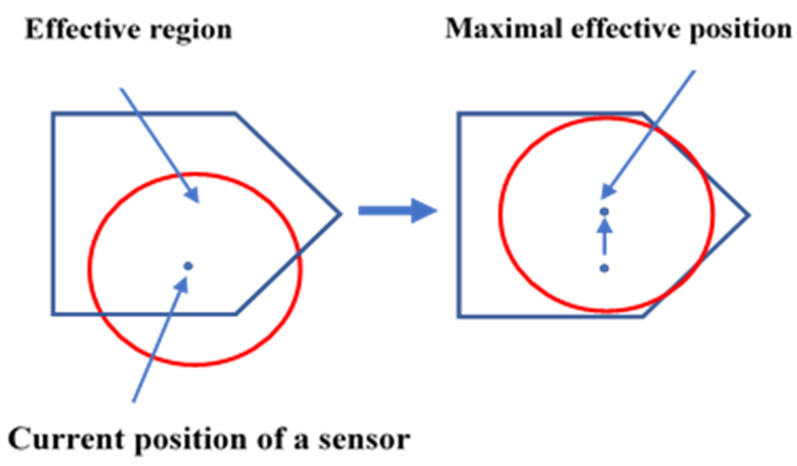
The effective region and the maximal effective position.

The primary objective of WSNs is to provide maximum coverage with a limited number of node deployments when the sensing area has unfavorable conditions. In most research, Computational Geometry has played a significant role in sensor deployment schemes in WSNs. One of the essential types of research on coverage hole detection and healing has been proposed in [22]. The research shows that the movement of sensors can be controlled by using two methods: the Basic Method and Virtual Movement. In the Basic Method, sensors move iteratively until they cover the maximum part of the coverage hole. On the other hand, there is no actual movement of sensors in Virtual Movement. Based on Virtual Movement, the final effect is calculated on the coverage hole; however, the real action takes place only if it covers the coverage holes. The movement of sensors becomes reduced, as only one movement is noticed at any given time. In both the activities, i.e., Basic and Virtual, three algorithms are commonly used: the Vector-based algorithm (VEC) from a dense area, where sensor nodes are pushed away; the Voronoi-based algorithm (VOR) where sensors move towards the coverage holes; and the Min–Max, where sensors move towards coverage holes without generating new holes. The authors in [23] have proposed a Voronoi Coverage Hole Discovery algorithm (VCHDA). This algorithm helps find a hole by calculating the distance between the sensor node vertex and the Voronoi cell’s edges. The algorithm can recognize coverage holes efficiently and even provide labels for the border nodes adjacent to the coverage holes. The authors in [24] have presented an energy-efficient scheme using attractive and repulsive forces for self-node deployment. In this scheme, every node of the confined Voronoi Polygon is assigned a sensing task. The overall coverage problem can be converted to a local coverage problem by using a constrained Voronoi Polygon, as depicted in Figure 9.

The attractive force is generated from the Centroid of the Voronoi Polygon, and the repulsive force is generated from the obstacle or the neighboring node. This scheme offers a solution for uneven node distribution and thus provides maximum coverage in a shorter timeframe with less node movement.

The authors in [25] have focused on improving the area coverage of the Directional Sensor Network (DSN) in accordance with the rotatable directional sensors. To solve the difficulty of field coverage in the case of DSN, the authors have used the Voronoi Diagram and implemented a distributed greedy algorithm to reach an optimal solution. The direction of sensors could be selected and adjusted with the help of three principles: to enlarge the sensing area as much as possible within the cell, to reduce the overlapping of coverage with neighbors, and to keep away the sensor coverage from outside the field of interest. To cover the coverage holes faster, the authors in [26] have introduced two new schemes to deploy the nodes efficiently. These proposed approaches are called the Centroid and Dual Centroid approaches based on the Centroid of Voronoi Polygon. In the Centroid Scheme, the sensor moves to the center of the local Voronoi Polygon. However, the Centroid of the neighboring node polygon is calculated in dual Centroid. Then, the sensor moves to the center point of the line created by the local Voronoi Centroid and the neighbor node Polygon Centroid. The calculated new value is further used to get the new location for node deployment to get better coverage.

**Figure 10:**
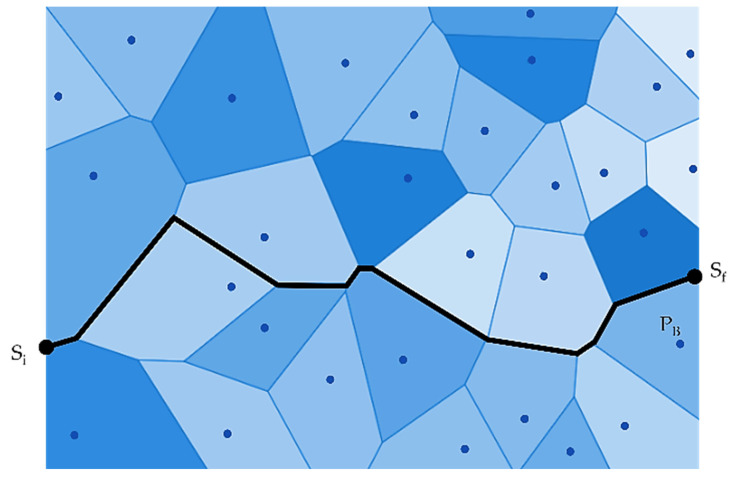
A maximal breach path (P_B_).

The authors in [27] have used Computation Geometry and Graph-Theoretic techniques. In Computation Geometry, the authors have used the Voronoi Diagram and later combined it with the Graph Search algorithm. The authors have also proposed that the “worst-case coverage-maximal breach path” and the “best-case coverage-maximal support path” algorithms can be used to find the optimal coverage in WSN. In the maximal breach path case, the Voronoi Diagram is used to deploy sensors, and then, subsequently, graph theory abstractions are used, as shown in Figure 10.

**Figure 11:**
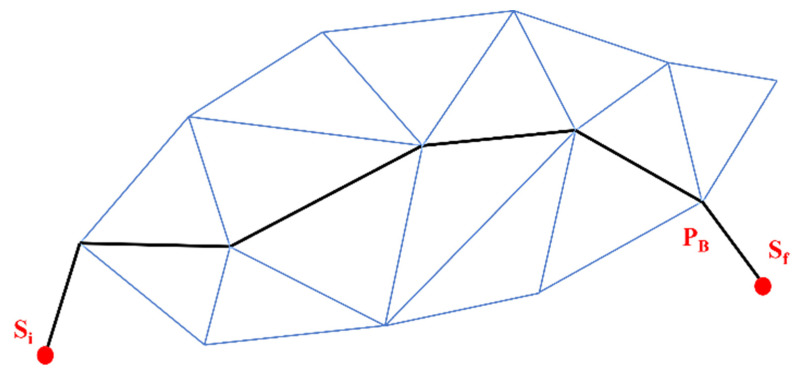
A maximal support path (PS).

On the other hand, in the maximal support path, the Voronoi Diagram is replaced with Delaunay Triangulation, and some weights are assigned according to line segments in Delaunay Triangulation. As explained in Figure 11, two additional edges are set up to connect S_i_ (initial location) and S_f_ (final place) adjoining sensors in the configuration. After deployment of a single node, results have shown a 10% improvement in coverage when 100 sensors are deployed randomly.

The authors in [28] have designed two novel schemes to solve the coverage problem. The Blind Zone Centroid-based method directs the sensor to the Centroid of the Voronoi Blind Zone Polygon’s Centroid rather than the Voronoi Polygon’s Centroid. The second proposed scheme by the authors is the Distributed Centroid-based system to enhance coverage in case the sensors are stagnated.

The authors in [29] developed a new coverage control method to ensure optimal coverage. To cover the region with changing boundaries, a blanket coverage area is used along the edge of the region. The newly proposed method provides a solution for moving coverage regions. The coverage area can be expanded or compressed according to the coverage domain. The authors in [30] have proposed an efficient self-deployment algorithm (ESA). The ESA describes the movement of a particular node towards the best position after initially putting it at a random position. Based on neighbor distance, the ESA identifies the accurate direction and distance from the existing sensor. This newly proposed approach gives healthier results than the Centroid, the Dual Centroid, the VOR, and the Min-Max algorithms. It has shown a significant improvement in the coverage and connectivity of WSN. The authors in [20] have resolved coverage issues by using the Voronoi Diagram. The authors avoided using the Global Positioning System (GPS) to corroborate the data for the construction of the Voronoi Diagram because of the cost factor and the lack of a guarantee of providing a solution in all the expected situations. They have proposed a region-coverage algorithm where GPS is not required for data corroboration. With the help of the Voronoi Diagram, a fully engaged sensor can be discovered.

The authors in [31] have explained how a Voronoi Diagram can be used to deploy sensor nodes that help inefficient energy utilization compared to the other available grid coverage strategies. A Voronoi Diagram is preferred over other geometrical data structures because its complexity is maintained by only one parameter, i.e., the total number of sensor nodes. This parameter uplifts effective energy consumption because that increases the network’s lifetime. The authors in [32] have proposed the concept of Prioritized Geometric Area Coverage to enhance the coverage in directional sensor networks. The proposed algorithm establishes a relationship between the cell area and the area covered by the sensor node during the selection of the sensor’s direction to maximize the coverage. The Voronoi Diagram marks the best sensor node as the node itself to cover the corresponding Voronoi-cell area. Also, the categorization has been carried out based on geometric shapes of the Voronoi cells formed. Also, the authors have focused on the network lifetime by switching off the redundant nodes. The authors in [33] have covered the issues of coverage and energy efficiency in the Voronoi-Glowworm Swarm Optimization K-means algorithm. The authors have used the K-means algorithm for cluster formation and cluster center selection in the proposed paper. The concept of the Voronoi cell is used to mark the appropriate sensing radius to obtain optimized coverage. A Glowworm Swarm Optimization is applied to decide the optimum location points to deploy the sensor nodes. Then, the active and sleep modes are used to utilize network energy efficiently. The authors improve coverage of heterogeneous WSN in [33] by using the coverage gap-fixing method with the help of the Voronoi polygons. The coverage gaps have been fixed with the help of Computational Geometry techniques and graph theory. The coverage holes created near the static nodes were defined and highlighted with the help of the Voronoi polygons. Redundant mobile nodes covered these holes. Table 2 gives a quick overview of the different Voronoi diagram-based techniques to improve coverage and connectivity in WSNs.

### 3.2. Delaunay Triangulation Based Techniques

**Figure 12:**
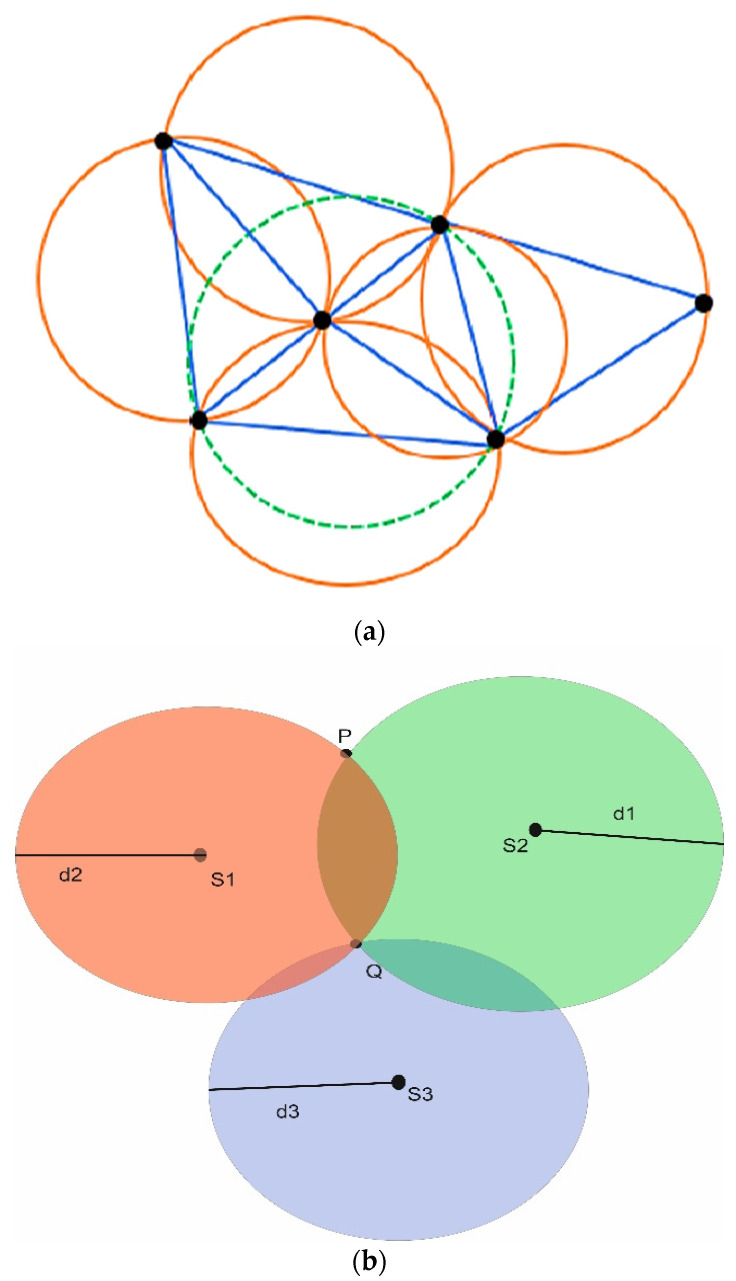
(**a**) Delaunay triangulation; (**b**) Trilateration.

Delaunay Triangulation (DT) is constructed by segmenting a specified area into several triangles so that the circumcircle of each triangle contains no points, as illustrated in Figure 12a. It is a kind of dual graph of the Voronoi Diagram for a discrete point set P. DT can be constructed by connecting the consecutive nodes in the Voronoi Diagram that share at least one standard edge. The two nearest neighboring sites can similarly be determined using the shortest edge in triangulation [34]. Trilateration is used for sensor node localization by using the geometry of a triangle, a circle, or a sphere with the help of neighboring nodes as shown in Figure 12b. Quadrilaterals or Polygons are formed by Trilateration made up of overlapping of triangles [35]. Trilateration is used to measure the side length of triangulation instead of the horizontal angle.

The Delaunay-based Coordinate-free Mechanism (DECM) was proposed by the authors in [36] to heal the coverage holes with less sensor movement by using a cooperative movement mechanism. The DECM is more efficient than other schemes for coverage hole detection and healing and even ideal nodes can also be addressed. In addition, it is a distributed scheme that prevents new hole generation during the healing process. The author in [37] has used Constrained Delaunay Triangulation (CDT) to solve the problem of coverage in WSNs. The CDT phenomenon optimally reduces the energy requirement by minimizing the information sent to the base station. In the concept of CDT, the author has focused on extra user-defined edges that do not form part of DT. Furthermore, CDT is also used to construct a set that contains the maximum number of nodes to supervise the area of interest.

The authors in [38] have discussed two major node deployment schemes: Random and Grid-based. In a Random deployment scheme, nodes are not deployed effectively as some nodes are kept in the same place while other locations remain uncovered. Triangular, Hexagon, and Square are the basic Grid-based deployment schemes. In the Grid-based deployment scheme, the nodes are placed regularly, but the deployment fields are kept regular. Hence, the author discussed a deployment method using DT by keeping all these issues in mind. For node deployment, firstly, the trusted region is divided into different triangles using the DT method, and then finally, the nodes are deployed on the triangle’s vertices.

**Figure 13:**
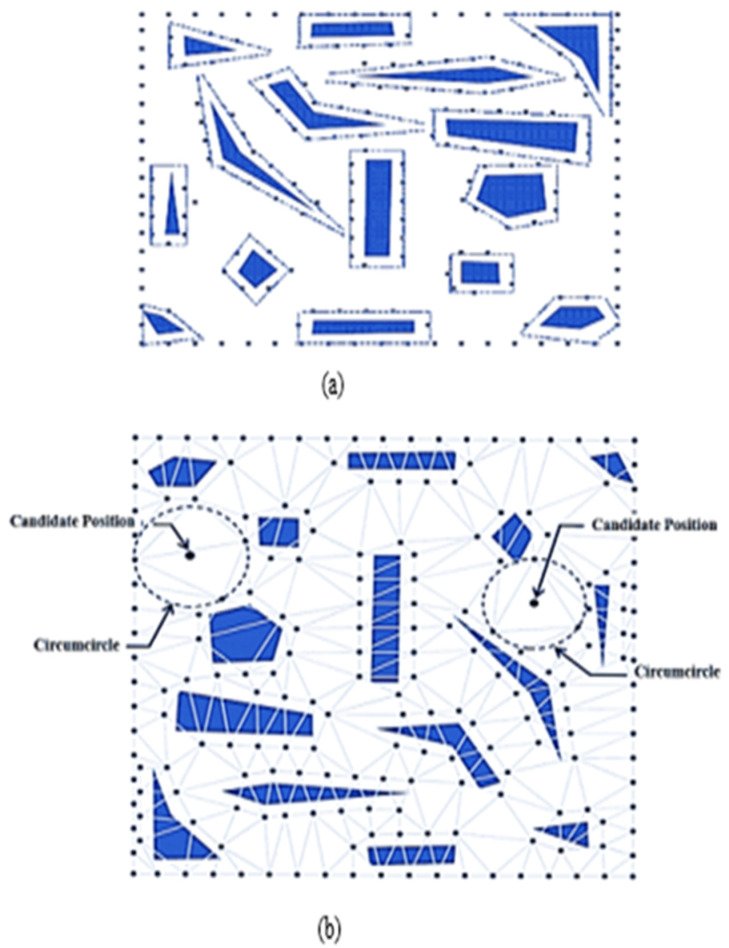
(**a**) The contour points generation step and (**b**) Delaunay triangulation of given sensors.

The authors in [39] have described DT in the 2D plane as a dual structure of the Voronoi Diagram. DT has satisfied the empty circle property, which is described as a circle passing through edge endpoints without containing other points. The DT empty circle property is used to find a suitable node deployment position. The two phases commonly used in DT are Contour Deployment and Refined Deployment. In the first phase, contour points are generated at the coverage area boundary and near the obstacles, in order to remove the coverage holes, as shown in Figure 13a. In the second phase, the node positions can be extracted by using DT for maximum coverage gain, as shown in Figure 13b. Finally, after determining all possible positions, the candidate with the best score is chosen for the final node deployment.

The authors in [40] have proposed a Delaunay-based Connected Cover (DBCC), which is a two-phase algorithm. The first phase focused on providing coverage over the network area when a tiny number of nodes were selected. Based on DT, the selection of the number of nodes is defined. In the second phase, the search algorithm is used to maintain the network connectivity breadth and more nodes are added subsequently. The shortest path between the connected components is determined to identify the subset of activated nodes when needed. All nodes selected for a particular path are switched on. The authors in [41] have used a dual DT to deploy nodes at each vertex of triangulation. The authors have also considered 3D surfaces for node deployment. The vertex positions are selected based on the sensing radius to gain maximum coverage. However, to find out the actual coverage rate, the issue of superposition is also handled. The same is checked to confirm whether the node deployed fulfills the communication requirements of WSNs.

The authors in [42] have used the basic idea of the Voronoi Diagram and DT. It has been used to make a cover set and a schedule cover set. The main goal is to cover the target area entirely by splitting the sensor into separate cover sets. Every sensor in a cover set must sleep or wake up at a desired time. The main focus is on finding a cover set so that the area covered by every sensor can be effectively minimized. These cover sets are scheduled for sleep and wake-up modes in order to enhance the network life. This is achieved by using a Geometric-based Activity Scheduling scheme (GAS). There are two phases in this algorithm: the Initialization phase and the Sensing phase. The sensors are scheduled for the wake-up and sleep modes by using the Initialization phase and the Sensing phase in continuum. The main aim of the GAS scheme is to minimize the number of active sensors at any given time.

Based on DT, the authors in [43] have described a new measurement scheme that gives essential information and correlates the distance between sensors—fat, thin, healthy—and areas between sensors. By using DT, this method provides a finer, more acceptable quality of service than other schemes that are only related to field coverage. DT is an optimal triangulation technique for a network having more than three sensors with the properties as described:

- For a given set of points, the outer polygon of the triangulation is convex;
- The triangle edges of each sensor connect it to its closest neighbors;
- Each sensor has a degree of at least two, if not three, sensors that are on the same shared straight line;
- There are no other sensors on the circumcircle of each triangle.

Four concepts explained by the authors which explicate Quality of Coverage (QoC) are:

■ The distance between each point in the field and its closest sensor is represented by the Probabilistic Distribution Function (PDF) (Coverage Resolution Model);
■ Coverage uniformity;
■ Areas that are perfect or scattered;
■ The distance between sensors with the emptiest space.

**Figure 14:**
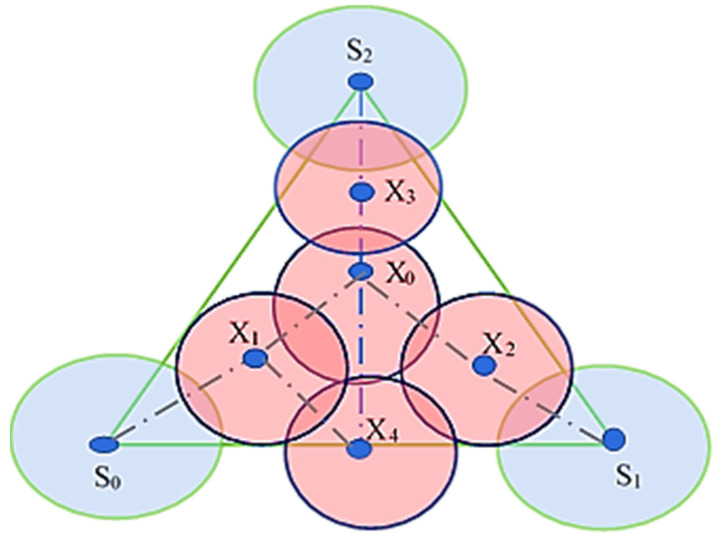
The position of assisted sensors.

The area coverage issue for the area of interest, which is of irregular shape, is resolved by the authors in [44]. The authors presented a DT-based method, namely the Heal Coverage Holes algorithm (HCHA), to discover and heal coverage holes. A hybrid sensor network has been used for area coverage. In this, both types of sensors are used, i.e., mobile and static sensors, as depicted in Figure 14. Static sensors are used to find the size of coverage holes, and aided mobile nodes are deployed to heal the coverage holes. This algorithm detects coverage holes and healing is subsequently carried out using DT.

**Figure 15:**
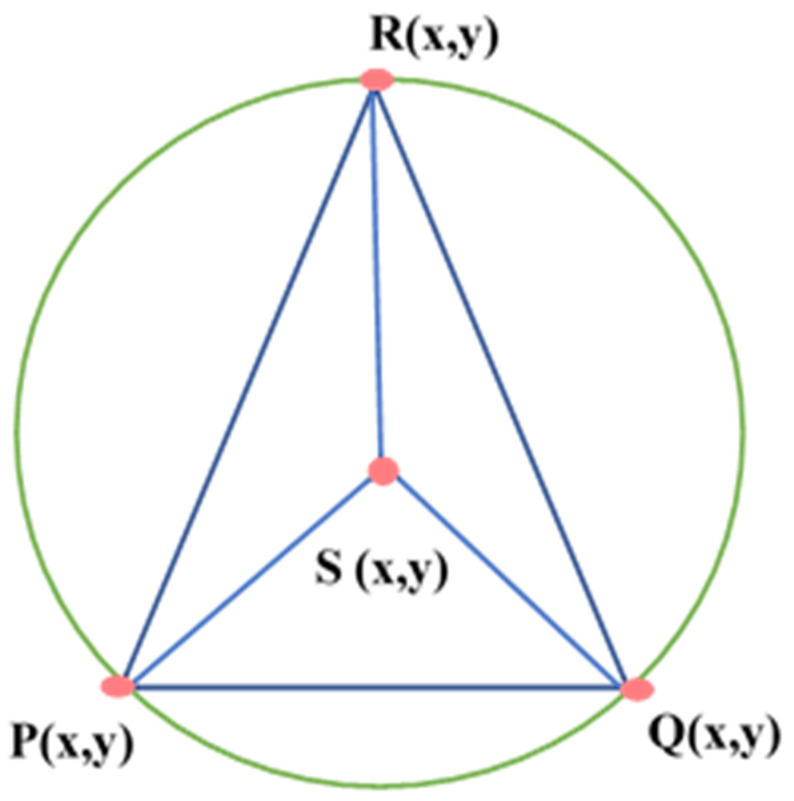
A representation of the empty circle property.

To detect coverage holes in a given area of interest, the authors in [45] have used a Coverage Hole-Detection algorithm (CHDAE). A DT with an empty circle property, as shown in Figure 15, was created to define the network topology. DT is a collection of points where the circumcircle of every triangle is empty. The authors have described three phases: (1) creating the Delaunay Triangle by using the approximation method to find the position of sensor nodes, (2) coverage hole detection in a comprehensive WSN, and (3) the estimation of the area of detected coverage hole, based on Computational Geometry.

A three-phase sink-and-sensors deployment strategy, the Evaluated Delaunay Triangulation-based Deployment for Smart Cities (EDTD-SC), is popular and outperforms random and regular deployments in terms of the area coverage and the end-to-end-delay by 29.6% and 29.7%, respectively [46]. The authors considered the indoor as well as the outdoor obstacles. The first phase is the configuration phase which includes a city map and obstacle information. The second phase is deploying the sensor nodes within the city based on the configuration phase. After deploying the sensor nodes, different triangles have been created using Delaunay Triangulation. The coverage evaluation step is defined to find the location of the new sensors. The coverage ratio helps in discovering the area with fewer sensors. It is calculated using the triangle’s center point. The third phase explained the proper position of sink deployment to gain a high network performance. The K- means clustering algorithm has been defined to make clusters based on the sensors deployment phase. Regarding area coverage and end-to-end delay, the EDTD-SC gives excellent results by considering the regular and the random deployment of sensor nodes. Various Computational Techniques based on Delaunay Triangulation have been described in Table 3.

### 3.3. The Voronoi Tessellation-Based Techniques

**Figure 16:**
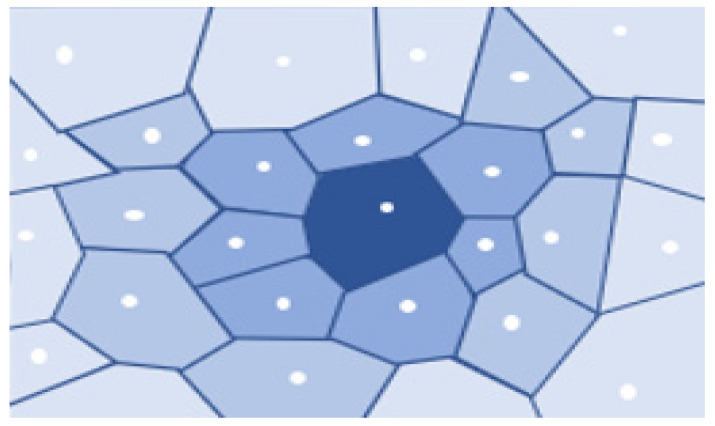
Voronoi tessellation.

Voronoi Tessellation is an approach that is used to divide the given region into n units to structure uniform and non-uniform meshes. Voronoi Tessellations have various applications in computer science, chemistry, and the social sciences, etc. The Voronoi partition monitors the collection of disjoint regions by using sensors. Figure 16 describes the basic idea of Voronoi Tessellation. As shown in Figure 15 the given region is divided into n number of cells to create a tessellation. White dots depict the sensor nodes residing within the specific area. Every cell consists of only one sensor node, which is nearest to every point in the cell as compared to other neighboring sensor nodes. One of the best algorithms to create Voronoi Tessellation is Fortune’s algorithm.

**Figure 17:**
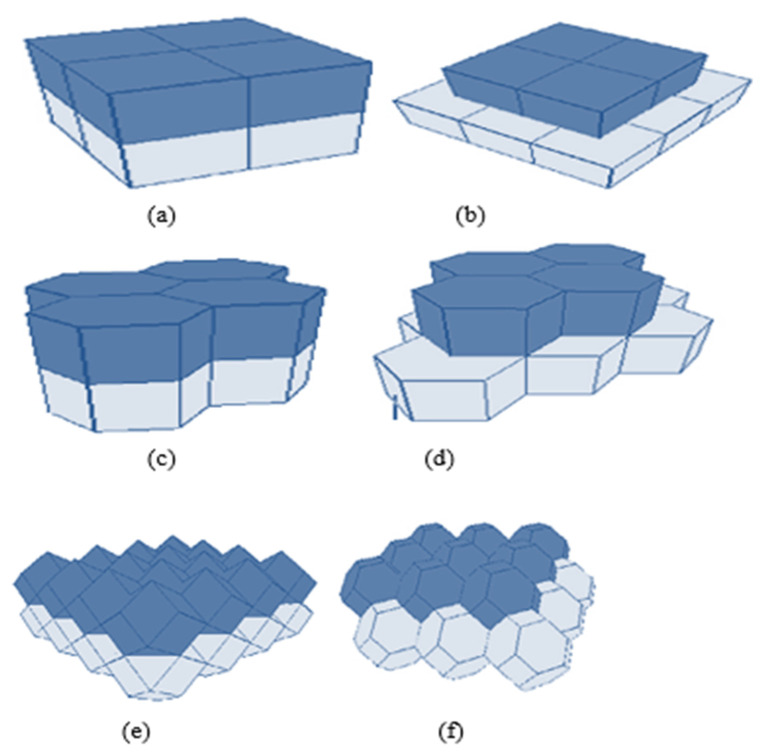
Different 3D space-partitioning shapes (**a**) a Cube, (**b**) an Alternative-cube, (**c**) a Hexagonal Prism, (**d**) an Alternative- hexagonal prism, (**e**) a Rhombic Dodecahedron, and (**f**) a Truncated Octahedron.

The authors in [19] have proposed a placement strategy based on the minimum number of nodes using Voronoi Tessellation. A truncated octahedron is used to cover the 3D sphere with a high volumetric quotient, as shown in Figure 17. A truncated Octahedron renders the best strategy compared to other data structures like the “Hexagonal Prism and Rhombic Dodecahedron.” The basic idea is taken from Kelvin’s conjecture, which explains: “What is the optimal way to fill a 3D space with cells of equal volume, so that surface area is minimized?”. Kelvin provided a solution with a truncated octahedron of 14 sides with isoperimetric inequality, but could not prove its optimality.

The authors in [18] have used two Voronoi Polyhedrons, a Truncated Octahedron and a Hexagonal Prism, to accomplish full coverage and 14-connectivity and 6-connectivity, respectively. The authors have considered a Truncated Octahedron lattice pattern for 14-connectivity, eight of its 14-connected neighbor sensors are placed at the cube’s corner, and six are placed at the center of the six neighbor cubes. A Hexagonal Prism is used to achieve 6-connectivity; therefore, sensors at the cube’s center have six connected neighbors. Different Grid-based node deployment strategies are proposed by the authors in [47], such as a triangle, a square, a hexagon, a pentagon, and an octagon in a 2-D space. The authors equated all strategies with a respective number of sensors and coverage areas. It is resolved by them and the results that the octagon has the highest coverage, but the triangle and the hexagon have the least range. Although the octagon has the highest coverage, it requires the maximum number of nodes. Comparing these coverage issues and the number of nodes required, it has been concluded that a square is the best and most practical scheme.

The authors in [48] have proposed a three-dimensional coverage pattern based on cuboids by analyzing the regular polyhedron model. The minimum number of the sensor ensures complete network area coverage. The sensor nodes are deployed in the area of interest in the finite 3D grid. The authors assumed all the sensors were perceived in a uniformly sized sphere-shaped sensing field. As a result, the need for complete coverage of the monitored area can be met. This deployment method effectively reduces the number of nodes compared to the traditional random way. The authors in [49] have developed a method based on Voronoi Polygons for boundary node detection. A cluster can be defined by two types of boundaries: outside and inside edges. The coverage boundary problem needs to be transferred to identify all boundary nodes and there is a sudden reply to change the coverage without placing any communication burdens. For WSN coverage boundary node detection, the authors have developed a deterministic and localized algorithm. The technique relies entirely on one-hop information and a few easy local computations.

The author in [50] has given the solution to the optimal polyhedron for 3D space filler, which maximizes the coverage of sensors. It is found that the great rhombi cuboctahedron is the single largest enclosed convex polyhedral space filler. A Sensor selection algorithm is proposed for deterministic sensor placement, wherein the sensor is placed at the Centroid in a non-overlapping polyhedral space filler. The authors in [51] have designed a redeployment algorithm to achieve full coverage in 3D space. Every consumption factor is also considered as the sensor nodes have minimal energy. The authors have extended the Voronoi partitioning of 2D space to 3D, optimized according to the 3D partitioning. The author proposed a three-dimensional distributed virtual forces algorithm (3D-DVFA) commonly used for target detection. The algorithm is based on virtual forces where every node has attractive and repulsive forces.

**Figure 18:**
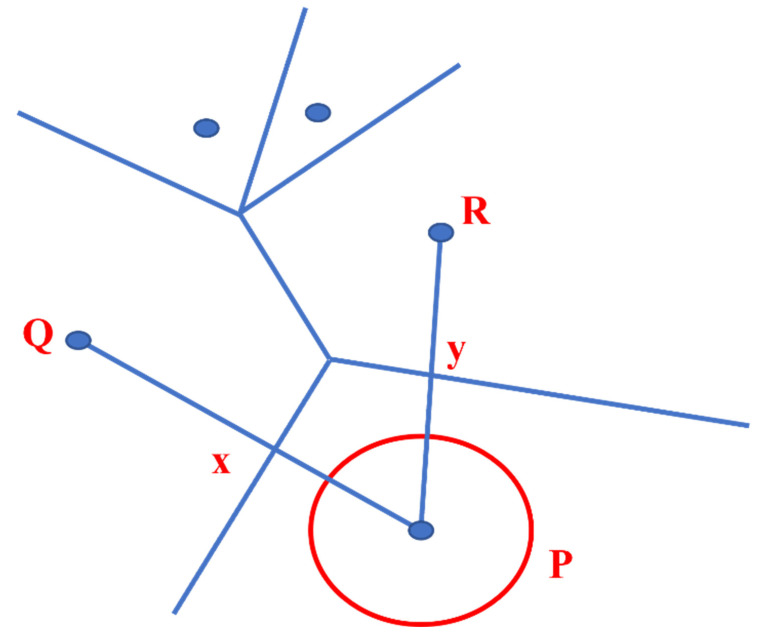
A simplified mobility model.

The K-Voronoi-based mobility model manipulates the coverage according to the target. Based on the Voronoi Diagram, a simplified mobility model is shown in Figure 18. This model enhances the overall coverage while maintaining more mobility choices. This mobility model preserves k-coverage by maintaining ground sensors in motion. These models allow multiple nodes to sense the area of interest simultaneously. The activity scheduling approach is also used to reduce the redundant nodes and save energy. The K-order Voronoi Diagram controls the sensor node mobility and the activity schedule [52].

The authors have proposed a heterogeneous sensor network in [53] to detect and heal holes in the coverage. The sensing radius of the new sensor deployed is different from the initially deployed sensors because some coverage holes of different sizes exist in the networks. The relationship between the radii of the nodes deployed initially, and the radii of nodes deployed later are explained to fill the various holes such as the trigonal, tetrahedral, octahedral, and interstitial holes. The sensors with the small radii have been deployed to fill the coverage hole and reduce the overall area’s cost. Table 4 expounds on different Voronoi Tessellation-based techniques to improve coverage and connectivity in WSNs.

### 3.4. The Convex Hull-Based Techniques

The smallest convex polygon containing a particular set of nodes is called the Convex Hull of ‘*n*’ nodes. Graham’s Scan algorithm can be used to construct it in *O* (*n* log *n*) time [54,55]. In the two-dimensional Convex Hull, the set of points, i.e., set *p* = {pt, t = 1, 2,3, …, *n*} on a 2D plane V, and any limited points at set *p*, form all of the convex grouping and then construct a set *H*, which is called the Convex Hull of set *p* and can be represented by *H*(*p*) [56]:

(1) $$H\left(p\right)=\left\{\sum_{i=1}^{m}\varepsilon_{i}x_{i}|x_{i}\in p,\varepsilon_{i}\geq 0,\dot{l}=1,2,\ldots ,m,\sum_{i=1}^{m}\varepsilon_{i}=1,m\in N\right\}$$

**Figure 19:**
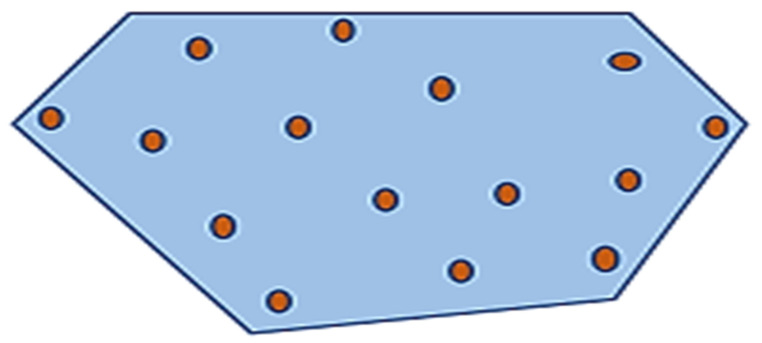
A 2D Convex Hull.

In a two-dimensional plane V, *H* is the smallest convex polygon encompassing all the points in the set *p* and can be thought of as a two-dimensional Convex Hull containing the set *p*. The same is illustrated in Figure 19, where the geometry is a Convex Hull surrounding the point set in solid lines.

**Figure 20:**
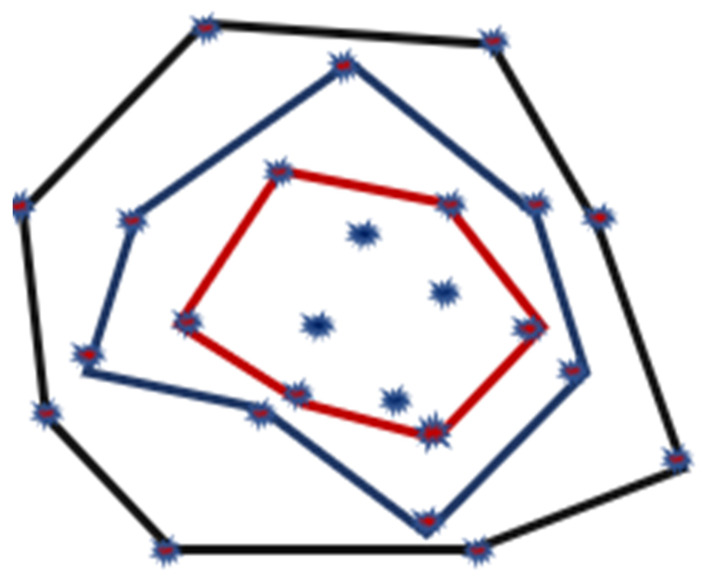
The Multi-layered Convex Hull set.

**Figure 21:**
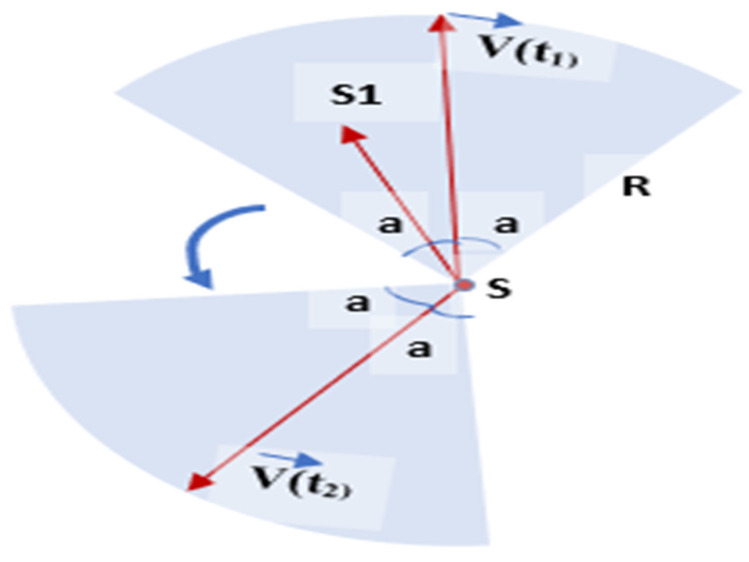
Sensing directions of directional nodes.

The minimum area convex set of a planar set having the original set known as the Convex Hull provides many solutions for existing problems. These problems are like Pattern Recognition, Image Processing, and Computer Graphics, in which a set consists of either an ‘*n*’ sided polygon or a set of *n*-points. The authors in [57] offer a coverage enhancement approach for reducing the overlapping sensing areas encompassed by many neighboring directional nodes. The directional sensing model estimates the total number of directional nodes used for a given area coverage. A sensing-connected sub-graph effectively divides the sensing area into distributed components. To improve coverage, a multi-layered Convex Hull set is employed to simulate each sensor-connected subgraph, as illustrated in Figure 20. Firstly, each sensing-connected subgraph forms a multi-layered Convex Hull set. Then, the direction of directed nodes is rotated to acquire the maximum sensing coverage area, as shown in Figure 21.

The authors in [58] have proposed a source localization approach. When the source exists outside the Convex Hull, the authors examined the local convergence problem and the problem with the projection onto the convex set (POCS). A fast, low, complexity approach, i.e., a semi-definite programming algorithm, has been created based on a problem revolution for two different source localization models.

**Figure 22:**
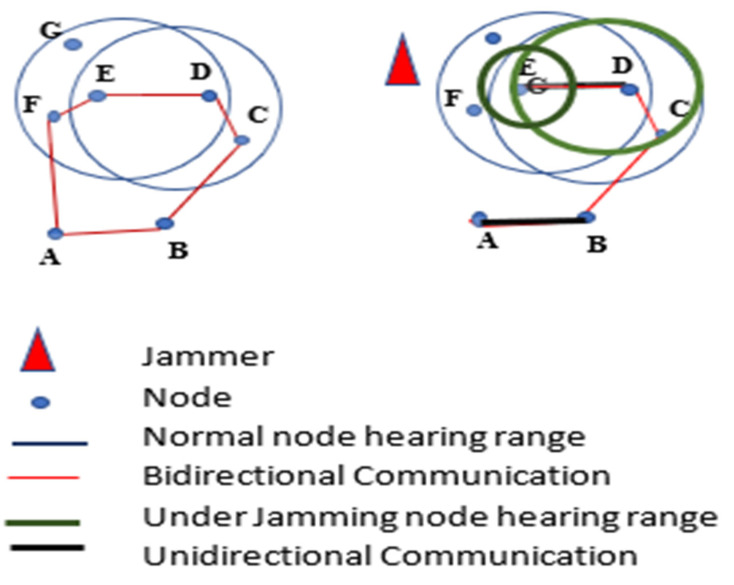
Jamming effects on WSNs.

**Figure 23:**
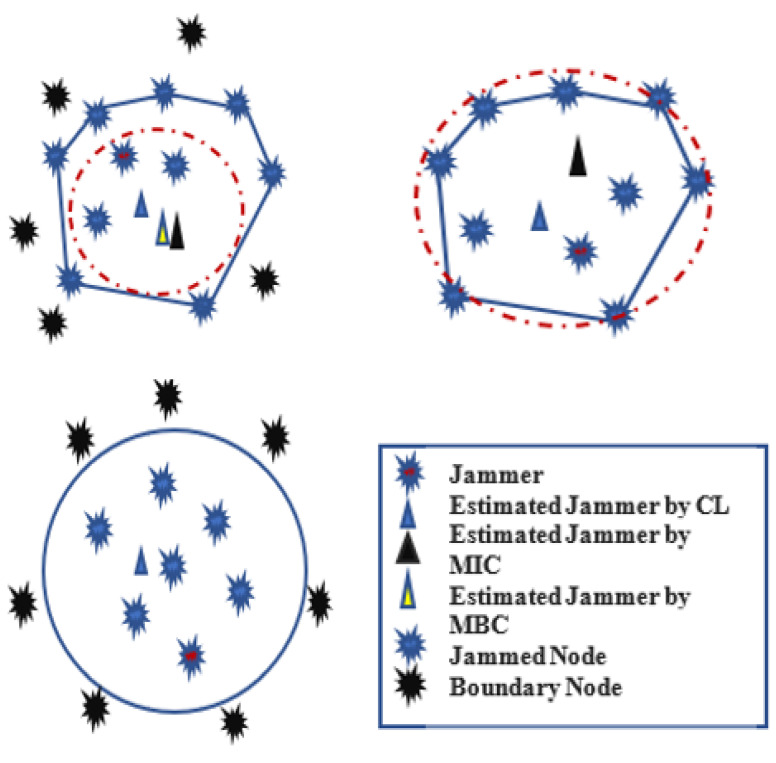
Double circle localization (DCL).

The authors in [59] proposed a Maximum Inscribed Circle (MIC) and Minimum Boundary Circle (MBC) -based approach called a Double Circle Localization (DCL) to address the problem of jammer localization. Figure 22 depicts the jammer model and the impacts of jamming on communication. Figure 23 shows the algorithm with no other assumptions regarding the crowded area. Different existing algorithms like Centroid Localization (CL), Weighted Centroid Localization (WCL), and Virtual Force Iterative Localization (VFIL) have been compared with the new algorithm DCL, which performs better in all proposed conditions.

The authors use the Convex Hull in [60] to solve the minimum path data gathering problem. This research selects a delegation node in every sub-network and then the Convex Hull-based algorithm is designed. Two properties have been used: (1) the Convex Hull boundary nodes must be present in the optimal solution of the Euclidean traveling salesman problem, and (2) the Convex Hull never intersects with itself. As compared to the greedy algorithm, the Convex Hull is closer to the optimal solution, and its complexity is *O*(*N* + *n*^3^).

The authors [61] presented the Voronoi Diagram Greedy (VD-GREEDY) and the Convex Hull Most Forward Progress within the Radius (CH-MFR) routing algorithms to address the twin issues of routing and geocaching. The VD-GREEDY is predicated on identifying node neighbors close to a potential target location and several methods have been discussed for determining such neighbors. The CH-MFR has described the Convex Hull problem on neighboring nodes, and the range directional (R-DIR) is a modified version of the presented directional methods. Loop avoidance, success rates, and flooding rates are the advantages of using CH-MFR and VD-GREEDY schemes. The authors in [62] have described a Second-Order Cone Programming (SOCP) -based two-step method for distributed localization. Based on limited information, the sensor nodes initially find their location, and subsequently, the anchor nodes upgrade their positions using information from neighbors. SOCP is used because of its straightforward structure and ability to solve problems faster. This technique improves the position of the anchor nodes in their neighbors’ Convex Hulls. In [63], two approaches are explored to resemble the forest fire forms with shared WSNs that work in a fully distributed manner without a base station and with the information collected by each node through broadcast. In the first approach, the punctual model is considered in which every node stores and forwards all information as received. This approach requires the distribution of a large number of data packets. To handle such a massive quantity of data packets, the Convex Hull-based model has been used. The nodes process all the information collected and discard what is irrelevant.

Consequently, each network node would be aware of the set of points reached so far by the fire at any given time. An interpretation of the fire processed with this data would be fairly true. In-network, the data aggregation technique is applied as the second approach to obtain a more compact fire model. The authors in [64] provide the geographic routing technique k-MLP (Multi-Lane Routing Path) to address caverns around a complex-shaped coverage hole. This scheme extends the network lifetime and the load balancing compared to other existing routing algorithms. The routing scheme consists of two phases: (i) the initialization phase and (ii) the routing phase. The boundary nodes inside a cavern compute the Convex Hull of the hole shape. The shortest routing path is determined in the routing phase with the idea of achieving almost k parallel lines. The authors in [65] have tried to reduce the overall cost of network establishment. For this, the authors have focused on the locations of node placement and trajectory determination for Mobile Sink (MS) so that targets are under the coverage of sensors and information packets are transferred to MS. Also, many studies resolve wireless sensor networks using artificial intelligent techniques such as in [66,67,68,69,70].

Moreover, the authors have utilized the concept of the convex polygon in the 2D plane and a graph-based approach to optimize the number of sensor nodes. Thus, reducing the total cost of network deployment. An overview of the different Convex Hull-based techniques is given in Table 5.

## 4. Current Research Challenges and Solutions

After knowing the sensor nodes’ positions, Computational Geometry approaches can be used to address several challenges in WSNs. Using the idea of CG Techniques, a number of sensor nodes can be optimized, and the longevity of WSNs can be improved to a certain extent. Due to the nature and deployment of WSNs, the creation of coverage holes is inherent, and these coverage holes can be detected and healed using CG Techniques. A summary of various problems that have been addressed and the corresponding contribution of various articles included in this study is presented in Table 6. Also, Table 7 which is showing the research issues/challenges which were covered or not covered (treated as limitations) is given in the same section.

## 5. Conclusions and Future Scope

This paper expounds on the existing work done in WSNs using Computational Geometry-based techniques. In comprising Wireless Sensor Networks, the arrangement of sensor nodes and their communication links, that is, network topology, plays a vital role. The structural properties of various Computational Geometric structures can be applied to network topology control. Computational Geometry is very helpful to maintain network connectivity and to optimize its lifetime and throughput, so that an efficient routing for the network can be designed. Furthermore, a network is fault tolerant only if it is *k*-connected. By merely employing various data structures, it has been made possible to solve recurring complications like fault tolerance, dimensionality, the discovery and healing of the coverage holes, the minimization of the number of node deployments, and the cutting down on costs, etc. Various optimization techniques have been examined to conclude the world-beating solution to overcome the existing loopholes. The proposed solutions would aid curious researchers in exploring existing data structures of Computational Geometry. During detailed analysis of actual work done, it has been ascertained that the research using the 2D computational models has been comprehensive. However, to bridge the dimensionality gap, it is pertinent to mention the 3D models as they simulate the real operating environment. The 3D savvy models would also attract the inherent challenges embedded in 3D computational models. In addition, techniques for reducing the number of nodes deployed for particular WSNs, while maintaining the coverage and connectivity, also draw the attention of research scholars. Hence, future aspirants need to be future-ready with the required aptitude to address these burning issues. On the basis of research articles included in the present work, some of these current issues are listed below-

(a) Future researchers should focus on working with the two-dimensional, and specifically the three-dimensional, heterogeneous environments where the sensing and communication ranges or the deployment of sensors with different sensing ranges may be different, etc.
(b) Researchers must direct attention towards achieving k-Coverage where k ≥ 1 in sensitive regions.
(c) Research should be performed within an environment where obstacles are present, as obstacles are always present in real scenarios.
(d) Researchers should also focus on issues like mobility of target events as well as the testing of sensors in physical environments.
(e) Researchers should try to work out the techniques for finding and repairing the coverage holes without creating new holes.
(f) Researchers should also address the issue of finding the shortest path for sensor node movement.

## Figures and Tables

**Table 1:** The Main Information Needed to Perform Scientometric Analysis.

Description	Results
MAIN INFORMATION ABOUT DATA	
Time span	2004:2022
Sources (Journals, Books, etc.)	104
Documents	208
Average years from publication	7.07
Average citations per document	17.62
Average citations per year per doc	2.22
References	6077
DOCUMENT TYPES	
Article	208
DOCUMENT CONTENTS	
Keywords Plus (ID)	1436
Author’s Keywords (DE)	540
AUTHORS	
Authors	559
Author Appearances	682
Authors of single-authored documents	7
Authors of multi-authored documents	552
AUTHOR COLLABORATION	
Single-authored documents	7
Documents per Author	0.372
Authors per Document	2.69
Co-Authors per Documents	3.28
Collaboration Index	2.75

**Table 2:** An Overview of the Different Voronoi Diagram-Based Techniques to Improve Coverage and Connectivity in WSNs.

Year/Reference	Strategy Used to Deploy Nodes	Node Type	Algorithm	Scheme	Space	Goal (Relevant to Question No.)	Coverage/Connectivity Enhancement	Simulator
2007/[20]	-	-	Localized Hole Discovery algorithm	-	2D	To provide a solution for hole discovery and fully sponsored sensor problem (Q5.)	Coverage Enhancement	-
2006/[22]	Random	Mobile	VEC, VOR, MIN-MAX	Distributed	2D	Hole Discovery and Healing (Q1., Q5. & Q3.)	Coverage Enhancement	NS2
2016/[23]	Random	Static and Mobile	VCHDA (Coverage Hole Discovery algorithm on Voronoi Diagram)	Semi distributed	2D	Coverage Hole Discovery and to identify the Border Node of coverage Holes (Q5., Q3. & Q7.)	Coverage Enhancement	MATLAB
2012/[24]	Random	Mobile	Centroid Directed Virtual Force	Distributed	2D	Self-Deployment of Nodes (Q1.& Q3.)	Coverage Enhancement	-
2014/[25]	Random	Mobile	IDS, IDA, OFCA	Distributed	2D	Coverage maximization of directional Sensor Network (Q2.& Q3.)	Coverage Enhancement	-
2009/[26]	Random	Mobile	Centroid, Dual Centroid	Distributed	2D	Self-Deployed Scheme (Q1. & Q3.)	Coverage Enhancement	-
2005/[27]	Random	-	Maximal Breach Path, Maximal Support Path	Distributed	2D	To solve the worst- and best-case coverage (Q1.)	Coverage Enhancement	-
2018/[28]	Random	Mobile	Blind Zone Centroid Based Scheme (BCBS), Distributed Centroid Based Scheme (DCBS)	Distributed	2D	To maximize sensors coverage (Q1. & Q3.)	Coverage Enhancement	-
2017/[29]	Random	Mobile	-	Distributed	2D	To provide optimal coverage for the dynamic region (Q1. & Q3.)	Coverage Enhancement	-
2020/[32]	Random	Mobile	Prioritized Geometric Area Coverage (PGAC)	-	2D	To enhance network lifetime (Q1. & Q3.)	Coverage Enhancement	PYTHON
2021/[33]	Random	Mobile	Voronoi-Glowworm Swarm Optimization K-means	Centralized	2D	To improve coverage and network lifetime (Q1. & Q3.)	Coverage Enhancement	MATLAB

**Table 3:** Overview of Different Delaunay Triangulation-Based Techniques to Improve Coverage and Connectivity in WSNs.

Year/Reference	The Strategy Used to Deploy Nodes	Node Type	Algorithm	Scheme	Space	Goal (Relevant Question No.)	Coverage/Connectivity Enhancement	Simulator
2014/[36]	Random	Mobile	DECM (Delaunay- Based Coordinate- Free Mechanism)	Distributed	2D	To detect coverage hole and find the shortest path for node movement to heal the holes (Q1., Q3. & Q5.)	Coverage Enhancement	GENIOrbit Testbed
2013/[37]	Deterministic	Static	Distributed greedy algorithm	Centralized	2D	To improve the quality of service by maintaining energy-constrained (Q1. & Q6.)	Coverage Enhancement	MATLAB
2013/[38]	Deterministic	-	Watson’s algorithm	-	2D	To improve Area Coverage (Q1.)	Coverage Enhancement	-
2007/[39]	Deterministic	Static	Score algorithm DT-Score algorithm	Centralized	2D	To improve the coverage of AOI with an obstacle (Q1. & Q4.)	Coverage Enhancement	-
2017/[40]	Random	-	(DBCC) Delaunay-based connected cover	Distributed	2D	To maintain minimum nodes cover set to preserve connectivity (Q1.)	Connectivity Enhancement	NS2
2017/[44]	Random	Static and Mobile	Heal Coverage Holes algorithm	Distributed	2D	To enhance coverage by finding and healing the coverage holes (Q1., Q3. & Q5.)	Coverage Enhancement	MATLAB
2018/[45]	Random	-	CHDAE (Coverage Holes Detection algorithm)	Distributed	2D	Detection of coverage holes and holes area estimation (Q1. & Q5.)	Coverage Enhancement	MATLAB
2020/[46]	Random	-	EDTD-SC	Centralized	2D	To improve coverage and strengthen connectivity (Q1.)	Coverage and Connectivity Enhancement	PYTHON

**Table 4:** An Overview of Different Voronoi Tessellation-Based Techniques to Improve Coverage and Connectivity in WSNs.

Year/Reference	The Strategy Used to deploy Nodes	Node Type	Algorithm	Scheme	Space	Goal (Relevant Question No.)	Coverage/Connectivity Enhancement	Simulator
2019/[1]	Random	Mobile	Improved Virtual Force algorithm	-	3D	To improve network coverage (Q1. & Q3.)	Coverage Enhancement	MATLAB
2015/[19]	Random	-	Kelvin’s and Kepler’s Conjecture	Distributed	3D	Full area coverage (Q1.)	Coverage and Connectivity Enhancement	Senetest 2.0
2017/[50]	Deterministic, Random	Mobile	Sensor Selection algorithm	Centralized	3D	To find an optimal polyhedron that maximizes the coverage quality of sensors (Q1. & Q3.)	Coverage and Connectivity Enhancement	High level the simulator is written in C
2015/[51]	Deterministic	Mobile	3D-DVFA (Distributed Virtual Force algorithm)	Distributed	3D	Self-deployment of nodes by ensuring full coverage (Q1. & Q3.)	Coverage Enhancement	NS3
2009/[52]	Random	Mobile	Advanced Voronoi-Based Mobility Model (AVBMM)	-	2D	To discover redundant nodes and reduce energy consumption (Q1., Q3. & Q6.)	Coverage Enhancement	MATLAB

**Table 5:** An Overview of the Different Convex Hull-Based Techniques to Improve Coverage and Connectivity in WSNs.

Year/Reference	The Strategy Used to Deploy Nodes	Node Type	Algorithm	Scheme	Space	Goal (Relevant Question No.)	Coverage/Connectivity Enhancement	Simulator
2006/[57]	Random	Static	Coverage Enhancing algorithm	Distributed	2D	To maximize network area coverage (Q1.)	Coverage Enhancement	Senetest 2.0
2008/[58]	-	-	SDP (Semidefinite Programming)	-	mD	To address the local convergence problem	-	MATLAB
2012/[59]	Random	NA	Double Circle Localization	-	2D	To localize Jammers	-	MATLAB
2009/[60]	Deterministic	Mobile	MPDG (Minimum Path Data Gathering)	Centralized	2D	To minimize the path length of the mobile mule (Q1. & Q3.)	Connectivity Enhancement	-
2006/[61]	Random	Static and Mobile	VD-Greedy, CH-MFR, and R-DIR	-	2D	To minimize the number of messages sent to find the position of the destination (Q1. & Q3.)	-	VC++
2007/[62]	Random	Mobile	SOCP (Second Order Cone Programming)	Distributed	2D	To solve the Localization Problem by reducing the size of the problem	-	MATLAB
2011/[63]	Random	Not Applicable (NA)	ABBA (Area-Based Beacon less Algo)	Distributed		To find the shape of the forest fire	-	NA
2021/[64]	-	NA	K-MLP (Multi-Lane Path Routing)	Centralized	2D	To find the shortest path between the source and destination (Q1.)	Extend network lifetime	Castalia
2021/[65]	-	Static and Mobile	Graph-based Approach	Centralized	2D	To reduce the overall cost of the network (Q1. & Q3.)	Coverage and connectivity Enhancement	-

**Table 6:** Summary of various Problem Statements and the corresponding solution/contribution of by various articles.

Year/Reference	Addressed Problem Statement	Main Contribution
2019/[1]	What is the efficient manner to detect a target in a three-dimensional coordinate system?	The 3-D Voronoi Partitioning Coverage algorithm is proposed to provide reliable results for target detection.
2009/[18]	How can a minimum number of sensor nodes in 3D space be deployed to achieve full volume coverage and k-connectivity?	Suggested a full-coverage design with (14 and 6 connectivity) using the Voronoi Polyhedron by implementing the Truncated Octahedron and the Hexagonal Prism.
2015/[19]	How can the number of nodes in the 3D area of interest be reduced?	It is proposed to use a Random node placement approach that incorporates a Truncated Octahedral to re-align nodes in the center of each cell created by Voronoi Tessellation.
2007/[20]	How can a Voronoi Diagram without any global location information be constructed?	The Centralized Construction algorithm is used to construct a Voronoi Diagram without any help of GPS.
2006/[22]	How can the required coverage be gained by estimating the right place of mobile sensor nodes?	Designed two sets of distributed protocols using the Voronoi Diagram: one protocol controls movement, and the other support communication.
2016/[23]	What is the way to label the border node of coverage holes?	Suggested a semi-distributed Coverage Hole Discovery algorithm through the use of the Voronoi Diagram.
2012/[24]	What is the optimal self-deployment scheme in Wireless Sensor Networks?	An energy efficient scheme using attractive and repulsive forces generated from the centroids of the Voronoi Polygon is designed.
2014/[25]	What is the optimal approach to improve the coverage in a directional sensor network?	A distributed greedy approach is used, which utilized the features of the directional adjustable sensors and the Voronoi Diagram to improve the coverage of the network.
2009/[26]	How can maximum coverage by healing the existing coverage holes be gained?	A self-deployment methodology using the Voronoi Diagram in addition to the Centroid and Dual Centroid schemes is proposed.
2005/[27]	How can optimal coverage in a wireless ad-hoc sensor network be achieved?	Two coverage algorithms are forethought to have a workable solution in hand: the “worst-case coverage for maximal breach path” and the “best-case coverage for maximal support path.” A Voronoi Diagram can be used prior to combining it with the graph search algorithm.
2018/[28]	How can a specific target position for a sensor to heal existing coverage holes be located?	Two novel schemes are designed to solve coverage problems based on two strategies, i.e., the Centroid-Based and the Distributed Centroid-Based.
2017/[29]	How can coverage and connectivity of an area with varying boundaries be controlled?	Voronoi-based approach is provided with the idea of agent moment towards a Voronoi cell.
2020/[32]	How can the working direction of sensor nodes to maximize the coverage and network lifetime be decided?	The categorization of Voronoi cells is carried out in order to minimize the overlapping area between adjacent cells.
2021/[33]	How can the sensor nodes be efficiently deployed in order to obtain optimized coverage with minimum energy consumption?	The concept of the Voronoi-cell is used to decide the efficient deployment of sensor nodes in the network formation.
2013/[36]	What is the effective way to heal the coverage holes by minimal sensor node movement?	The Delaunay-based Coordinate Free Mechanism is proposed to find the shortest path for nodes movement and for preventing generation of new coverage holes.
2013/[37]	How can fault tolerance in Wireless Sensor Networks be achieved?	Constrained Delaunay Triangulation is proposed to achieve fault tolerance and improve energy efficiency.
2013/[38]	How can an equal size division pattern of the target field to deploy nodes in a Grid-based scheme be achieved?	With Delaunay Triangulation, the area of interest is divided into different triangles and nodes are deployed on the vertices of the triangles.
2007/[39]	How can sensor nodes in the region with obstacles be deployed?	The Delaunay Triangulation Score method is used for deployment of sensor nodes.
2017/[40]	How can the number of nodes deployed be reduced while maintaining connectivity even with sensor nodes of limited energy sources?	A two-phase algorithm is used to minimize the number of nodes with minimum energy requirements.
2017/[44]	How can a region of interest be covered with less energy in effective way?	With the help of Delaunay Triangulation, the Heal Coverage Hole algorithm is used to provide full coverage.
2018/[45]	How can a coverage hole area in a region of interest be discovered with the help of the node’s location?	The empty circle property of Delaunay Triangulation is used to find the coverage hole area.
2020/[46]	What is a suitable way to determine the location of sink and sensor nodes in WSNs with obstacles?	With the help of Delaunay Triangulation, the location of the sink and sensor nodes is determined in the presence of indoor and outdoor obstacles.
2017/[50]	Observe the existence of connected coverage problems in 3D Wireless Sensor Networks.	A Rhombicuboctahedra is used as a 3D space filler to solve the problem of coverage and connectivity.
2015/[51]	What is the effective way to deploy nodes in such a way as to improve coverage and connectivity in the 3D network?	A redeployment algorithm using virtual forces is proposed to gain full coverage and connectivity by using Regular Dodecahedron Tessellation.
2009/[52]	How can the non-uniform coverage with respect to the presence of the target be controlled?	Based on a higher order Voronoi Tessellation, a multi-target tracking scheme is proposed.
2006/[56]	How can the coverage in the case of a directional sensor network be improved?	Sensing Connected Subgraph (SCSG) is used with the concept of the Convex Hull to improve coverage in the directional sensor network.
2008/[58]	How can the existing issues of WSNs, including source localization and tracking problems be addressed?	Based on the idea of min-max approximation to optimal maximum, a low complexity semi-definite programming (SDP) is proposed to utilize a Convex Hull.
2012/[59]	How can the location of a jamming device be found?	Based on the minimum boundary circle and the maximum inscribed circle, the Double Circle Localization algorithm is proposed.
2009/[60]	How can the path of a mobile node be minimized in order to get information from other nodes, when a network is subdivided into different subnetwork?	A Convex Hull-based algorithm is used to solve the problem of minimum path data gathering.
2006/[61]	How can the geo-casting and routing issues in Wireless Sensor Networks be solved?	The Voronoi Diagram Greedy algorithm and the Convex Hull Most Forward Progress within the radius routing algorithm are used to solve these issues.
2007/[62]	How can the problem of inaccurate anchor node position and the noisy distance problem be solved?	With the help of the Convex Hull, Second Order Cone Programming-based approach is used for this solution.
2011/[63]	What is the best way to find out the shape of Forest Fire?	An approach based on the Convex Hull is used to find the shape of the fire without any help from the base station.
2021/[64]	How can a complex shape coverage hole be efficiently covered?	A load-balancing K-Multi Lane Path Routing algorithm and the Convex Hull concept is used.
2021/[65]	How can the overall cost of maintaining coverage and connectivity be reduced?	The Voronoi Convex Polygon and Graph-based Approach is used to reduce overall cost.
2021/[71]	What is the effective way to reduce the wastage of existing resources to heal the coverage holes?	A Voronoi polygon-based coverage gap- fixing algorithm is applied to heal coverage holes with the help of already deployed nodes.

**Table 7:** The research issues/challenges covered or not covered (limitations) by various articles.

Reference	Research Issues or Challenges						
	Fault Tolerance	Environment				Efficient Resource Utilization	Boundary Coverage
	k-Connectivity	Homogeneous	Heterogeneous	Mobility	Presence of Obstacles		
Voronoi Diagram							
[20]	×	✓	×	×	×	✓	×
[22]	×	✓	×	✓	×	✓	×
[23]	×	✓	×	✓	×	✓	✓
[24]	×	✓	×	✓	✓	✓	×
[25]	×	×	✓	×	×	×	×
[26]	×	✓	×	✓	×	×	×
[28]	×	✓	×	✓	×	×	×
[29]	×	-	-	✓	×	×	×
[32]	×	✓	×	×	×	✓	×
[33]	×	✓	×	✓	×	✓	×
Delaunay Triangulation							
[36]	×	-	-	✓	×	✓	×
[38]	×	-	-	×	×	×	×
[39]	×	✓	×	×	✓	×	✓
[40]	-	✓	×	-	-	-	-
[44]	×	✓	×	✓	×	×	✓
[45]	×	✓	×	×	×	×	×
[46]	×	-	-	-	✓	-	-
Voronoi Tessellation							
[1]	×	✓	×	✓	×	✓	×
[19]	×	✓	×	✓	×	✓	×
[50]	×	✓	×	×	×	×	×
[51]	×	✓	×	✓	×	×	×
[52]	×	✓	×	✓	×	✓	×
Convex Hull							
[57]	×	✓	×	×	×	×	×
[59]	×	✓	×	×	×	×	×
[60]	×	-	-	✓	×	✓	×
[63]	×	✓	×	×	-	✓	×
[64]	×	✓	×	×	✓	✓	✓
[65]	×	✓	×	✓	×	×	✓

## Data Availability

Not applicable.
